# Observations on the Hooping Cough

**Published:** 1802-07

**Authors:** 

**Affiliations:** Preston


### *Observations on the Hooping Cough; communicated by* Dr. Bellamy, *of Preston.*

I Think it a duty I owe to Society, to communicate the ſucceſsful effects of any medicine, or mode of treatment for any diſeaſe, eſpecially for one whoſe ravages are ſo often fatal; I mean, mean, Hooping Cough; in recommending for which the artificial mufk, I am warranted by experiencing its powerful and permanent efficacy in the cafe of my infant daughter, who, a few months fince, laboured under that dreadful diftemper, previous to which the child had always enjoyed good health. The difeafe was ufhered in with common cold and cough, and had exifted about fix weeks before I began the artificial mufk. I had previoufly exhibited gentle emetics, frequently gave naufeating medicines, and kept the ftomach in a ftate of rejection. Opium and digitalis were alfo employed, both which merely palliated fo long as their immediate influence operated. Rhubarb with magnefia were occafionally given, to keep the bowels open. This mode of treatment merely feemed to keep the difeafe at bay; fond hope flattered from time to time, and with the infidious intermiffions of the complaint, feemed to lull me, as I believe is too often the cafe with others, into a kind of indolent expectation of fecurity by the work of time. I think it is too readily admitted that this, and moft other diforders, muft have their courfe; on the contrary, I feel fully perfuaded that it is the duty of a medical man ever to be active in new refources; and that that activity, and the power of the means he employs, fhould be proportioned to the action of the enemy he has to contend with. I do not mean to deny the operations of Nature in the cure of difeafe, nor am I incredulous of the fatality attending empirical practice; but, on the other hand, I have no doubt that as many lives are loft whilft we ftand thinking without acting, or rather whilft we do not think at all; but after having tried the old routine, refign the reft, like old nurfes, with a ftupid complacency, to Nature and a change of air; powerful agents, no doubt, but, alas! too often unavailing. This fact is too feverely felt by moft parents, and too well known to the profeffion at large not to be acknowledged. As in fome difeafes, it cannot in this be faid, that the mode of treatment ufually employed contributes to rid us of the patient. Death muft therefore follow from the power of the difeafe over that of Nature, and from the inefficacy of the common means effectually to affift her, or of taking the bufinefs fuccefsfully out of her hands.

It may be faid, I prefume too much from a fingle cafe; but I am fupported by the high authority of Profeffor Hufeland and the fuccefs of Mr. Bartley, in confequence of whofe Communications I was induced to employ the remedy in queftion, as the ne plus ultra; the cough returning, or rather increafing, with greater violence than ever, the child whilft in the fit bringing up blood by the mouth, and wafting in flefh and ftrength rapidly. (Though ferioufly alarmed for its fate, I certainly tainly cannot ftate it as the moft deplorable cafe.) I remembered having read the above gentlemens' accounts in your Journal, and turned to the pages and their advice immediately. I prepared the refinous fubftance, as directed by the Profeffor, and diffolved it in Sp. vin. R. inftead of alcohol, ufed by Mr. B. I began with three drops twice a day, and gradually increafed the dofe to fix, three times; laftly, I kept a little mixed in Aq. menth. c. magnes. alb. & mel. ord. which I gave a little of urg. tuff.

The relief obtained was really fudden and furprifing; indeed, the proportion of benefit was greateft in the firft twenty-four hours; the difeafe then abated, and the child was pofitively well of the cough in ten days; its health and fpirits returned as that went off, and in about a month were quite re-eftablifhed, and have continued fo to this day. One remark ought to be made, that ever fince, if a cough be excited by a crumb of bread, by crying, or other means, the characteriftic of the difeafe is evinced by a hoop or two, very fhort and trifling. This I confider to be the effect of fear and habit, (the powers of which are fo great even in infants; fear, indeed, of the acceffion of a fit, is to every obferver a painful mark attending the difeafe, and greatly augments its diftrefs) rather than the remains of the complaint, after four months; and, indeed, can be of no confequence in a picture of health.

I fhould have fent this cafe before, but expected to have been able to add others; which, however, the nature of my avocations as a Houfe Surgeon, has not afforded me an opportunity of doing. I have refolved to delay it no longer, hearing fo often of late the fatal effects of the difeafe. I hope others may be induced to give the artificial mufk a fair trial, and have no fmall confidence of their fuccefs.

*Feb.* 20, 1802.

P. S. On a future day, I may be induced to offer a few Remarks on the nature of the difeafe, the indication of cure, and the modus operandi of the artificial mufk.

