# Errata

**Published:** 1802-10-01

**Authors:** 


					Vol. viii. p. 41, 1. 4, from bottom* for Prefton^ read Oreiton.
?  P* 43? Houfe Surgeon, Vead Navy Surgeon.

				

